# Evaluation of mitochondrial function in chronic myofascial trigger points - a prospective cohort pilot study using high-resolution respirometry

**DOI:** 10.1186/s12891-018-2307-0

**Published:** 2018-10-30

**Authors:** Michael J. Fischer, Gergo Horvath, Martin Krismer, Erich Gnaiger, Georg Goebel, Dominik H. Pesta

**Affiliations:** 1 Vamed Rehabilitation Center Kitzbuehel, Kitzbuehel, Austria; 2 Department of Rehabilitation Medicine, Hanover Medical School, Hanover, Germany; 3 Department of Orthopedics, Medical University Innsbruck, Innsbruck, Austria; 4 Department of Medical Biochemistry, Semmelweis University, Budapest, Hungary; 5 D. Swarovski Research Laboratory, Department of Visceral, Transplant and Thoracic Surgery, Medical University Innsbruck, Innsbruck, Austria; 6 Department of Medical Statistics, Informatics and Health Economics, Medical University Innsbruck, Innsbruck, Austria; 7 Institute for Clinical Diabetology, German Diabetes Center, Leibniz Institute for Diabetes Research, Heinrich-Heine-University, Düsseldorf, Germany; 8 German Center for Diabetes Research (DZD), München-Neuherberg, Germany; 9 Department of Sport Science, University of Innsbruck, Innsbruck, Austria

**Keywords:** Mitochondria, Myofascial trigger points, High-resolution respirometry, Mitochondrial function, Muscle biopsy

## Abstract

**Background:**

Myofascial trigger points (MTrPs) are hyperirritable areas in the fascia of the affected muscle, possibly related to mitochondrial impairment. They can result in pain and hypoxic areas within the muscle. This pilot study established a minimally invasive biopsy technique to obtain high-quality MTrP tissue samples to evaluate mitochondrial function via high-resolution respirometry. Secondary objectives included the feasibility and safety of the biopsy procedure.

**Methods:**

Twenty healthy males participated in this study, 10 with a diagnosis of myofascial pain in the musculus (m.) trapezius MTrP (TTP group) and 10 with a diagnosis of myofascial pain in the m. gluteus medius (GTP group). Each participant had 2 muscle biopsies taken in one session. The affected muscle was biopsied followed by a biopsy from the m. vastus lateralis to be used as a control. Measurements of oxygen consumption were carried out using high-resolution respirometry.

**Results:**

Mitochondrial respiration was highest in the GTP group compared to the TTP group and the control muscle whereas no differences were observed between the GTP and the control muscle. When normalizing respiration to an internal reference state, there were no differences between muscle groups. None of the participants had hematomas or reported surgical complications. Patient-reported pain was minimal for all 3 groups. All participants reported a low procedural burden.

**Conclusions:**

This pilot study used a safe and minimally invasive technique for obtaining biopsies from MTrPs suitable for high-resolution respirometry analysis of mitochondrial function. The results suggest that there are no qualitative differences in mitochondrial function of MTrPs of the trapezius and gluteus medius muscles compared to the vastus lateralis control muscle, implying that alterations of mitochondrial function do not appear to have a role in the development of MTrPs.

**Trial registration:**

Registered as No. 20131128–850 at the Coordinating Center for Clinical Studies of the Medical University of Innsbruck, trial registration date: 28th November 2013 and retrospectively registered on 11th of October 2018 at ClinicalTrials.gov with the ID NCT03704311.

## Background

Myofascial pain syndrome is a leading cause of chronic musculoskeletal pain [1] with a lifetime incidence estimated to be up to 85% [2, 3]. It is characterized by local and referred pain, weakness, and restricted mobility [2]. Almost half of sick leave in the European Union is due to musculoskeletal disorders, which imposes a tremendous burden to healthcare resources [4].

Myofascial trigger points (MTrPs), a common and ubiquitous condition with (and cause of) myofascial pain [2, 5], were first identified by American researchers Travell and Simons, who described them as the dominant factor responsible for pain and functional limitations in the neuromusculoskeletal system [1]. MTrPs are palpable, taut bands found in stiff muscle that cause spontaneous pain (active MTrPs) or pain provoked by compression of the nodule (latent MTrPs) [2, 6]. This pain is often referred to other parts of the body, even in the absence of persisting nerve damage. Paresthesia, muscle weakness without primary atrophy, restricted mobility, proprioceptive disorders with impaired coordination, and autonomic reactions can also be caused by MTrPs [2, 6].

MTrPs are possibly caused by chronic overload, overstretching, or by direct trauma of the affected muscles [2, 5–7]. This can result in acute or chronic musculoskeletal pain, experienced by almost everyone during their lifetime. MTrPs have been found in 20–85% of the general population [8–11]. They can be treated holistically with stretching techniques, massage, pain medication, trigger point infiltration, dry needling, electrical stimulation, ultrasound, and cold laser treatment [2, 6, 12]. Myofascial pain syndrome can be initiated by the following events: damage to the sarcoplasmic reticulum, malfunction of the motor end plate, activation and sensitization of nociceptors [by adenosine triphosphate (ATP)], or the release of vasoneuroactive substances [5, 6, 13, 14]. The direct lesion of muscle fibers or persistently increased muscle tone are common factors related to the initial development of MTrPs [2, 5, 15].

The etiology of MTrPs is poorly understood. One of the earliest theories of trigger point formation states a continued shortening of the sarcomeres [6]. This is caused by extended calcium release from the sarcolemma due to abnormal endplate activity. ATP demand increases upon reuptake of calcium into the sarcoplasmic reticulum and induces relaxation of the muscle [13, 16]. Impairment of mitochondrial function due to a reduced cytochrome oxidase system stemming from a deficiency of freely accessible iron leads to an energy crisis within the muscle [17, 18]. Mitochondrial content determines the aerobic capacity of a muscle and is impaired in chronic musculoskeletal pain [18–22]. Lack of ATP propagates contracture and the resulting compressed capillary circulation can cause a hypoxic environment [1]. Data from respirometric studies on athletes, obese individuals, patients with diabetes or heart failure, and sedentary people indicate that hypoxia and ischemia can significantly affect and potentially impair mitochondrial function [23–35]. Inflammatory processes may also play a role as an increased concentration of inflammatory mediators including bradykinin, substance P, calcitonin gene-related peptides, tumor necrosis factor-alpha, and interleukins (ILs), such as IL-6, IL-1β, and IL-8 have been reported to be detected by in vivo microdialysis in MTrPs in humans [14].

In order to improve therapies and therapeutic tools for the treatment of MTrPs, understanding the mechanisms involved in their etiology is necessary. Elucidation of cell communication and signal transduction [15] or mitochondrial function from muscle biopsy samples to explore mechanisms at the level of the muscle cell are promising approaches. Based on the ‘energy crisis theory’ and disrupted mitochondrial energy metabolism in MTrPs, we assessed mitochondrial function in MTrPs in the present study.

The primary aim of this pilot study was to establish the clinical use of a minimally invasive biopsy technique to obtain high-quality muscle tissue from MTrPs in sufficient amount in order to evaluate their mitochondrial function via high-resolution respirometry. Secondary objectives included evaluation of the feasibility of the procedure in terms of patient acceptance and safety of the biopsy technique.

## Methods

### Study design and participants

In this prospective cohort pilot study using high-resolution respirometry to evaluate mitochondrial function in MTrPs, the primary endpoints were mitochondrial function expressed as oxygen flux (*J*O_2_; pmol O_2_.s^− 1^.mg^− 1^) and flux control ratios (FCR). Secondary endpoints to access the feasibility of the biopsy procedure in terms of patient acceptance were patient-reported pain, based on a Numeric Rating Scale (NRS) of 0–10, and patient-reported burden of procedure, based on a scale of 0–4, with 0 = extreme and 4 = none. Secondary endpoints to assess the safety of the procedure included: clinical wound assessment, consisting of assessing signs for local infection and inflammation (increased local temperature, swelling, redness and increased wound exudate), hematoma volume (assessed by ultrasound examination), and surgical complications.

This study took place at the Department for Rehabilitation Medicine of the General Hospital Hall in Tirol, Austria lasting from October 2013 through February 2014. The local television station for the province of Tyrol with approximately 50,000 viewers daily, ran a news documentary on myofascial pain and announced the study. Interested patients were advised to contact the principal investigator (PI), who determined their eligibility, obtained their informed consent, and enrolled them into the study. The study sample comprised 20 patients. Male patients aged 18–45 years with a clinical diagnosis of myofascial pain syndrome within the shoulder-neck muscles or the lumbogluteal region and the presence of an MTrP, defined as a firm palpation of a hard, tender nodule resulting in a spontaneous pain complaint [1], with symptoms present for 1 to 12 months were included. Exclusion criteria were:

- Signs that the participant’s prescriptive compliance was not expected (e.g., lack of cooperation)
- Disorders of the respiratory tract
- Neurological disorders, in particular neurodegenerative and neuromuscular diseases
- Disorders of the cardiovascular system or the musculoskeletal system
- Civil servants and military service personnel.

Those who met the inclusion criteria and additionally provided written informed consent were enrolled into the study. According to their specific pathology, participants were allocated to either a gluteus medius myofascial trigger point (GTP) or a descending trapezius myofascial trigger point (TTP) group with 10 participants in each group.

During the baseline visit, the participants’ demographic and anthropometric data were recorded, including weight, height, body mass index (BMI), type of sports practice, number of hours per week each sport was practiced, and smoking status. Each MTrP was assessed in terms of location, to determine if it was latent or active, and for pain. A MTrP was defined as being “active” if it caused spontaneous pain and referred pain pattern as described by Simons and Travell and as “latent”, if pain was provoked only by compression of the nodule [2, 6]. Patients reported pain following the compression of their trigger points [1]. Laboratory examinations were performed to analyze levels of C-reactive protein, creatine-kinase, and lactate dehydrogenase, as well as prothrombin time. Biopsies were obtained at baseline from GTP, TTP and the musculus (m.) vastus lateralis as control muscle, respectively, and analyzed as described below using high-resolution respirometry. Participants were asked to return to the study site for a follow-up visit 1 week after the biopsy.

During the follow-up visit, clinical wound assessment and an ultrasound examination was performed to determine hematoma volume. Surgical complications were reported and treated. Patient acceptance was assessed based on patient-reported pain (spontaneous pain at the trigger point without compression) and the burden of the biopsy procedure.

### Muscle biopsy sampling

Prior to performing the study procedures, the PI, an experienced surgeon who previously performed over 100 muscle biopsies on patients with neuromuscular disorders, was trained on the study biopsy procedure, which involved performing 10 biopsies (as described below) on a freshly slaughtered pig.

Local anesthesia was applied to the superficial skin covering the MTrP of each participant. Percutaneous biopsy sampling [36] optimized with a suction-enhancement technique was used to obtain muscle biopsies of the m. trapezius MTrP or the m. gluteus medius MTrP from each participant, using a small Bergstrom muscle biopsy needle, 8 swg (4.0 mm) × 100 mm (Dixons Surgical Instruments, Essex, United Kingdom). Biopsies were also obtained from the m. vastus lateralis of each participant to serve as a control sample.

Each muscle specimen was immediately placed in ice-cold biopsy preservation solution (BIOPS) containing 2.77 mM CaK_2_EGTA (ethylene glycol traacetic acid) buffer, 7.23 mM K_2_EGTA buffer, 0.1 μM free calcium, 20 mM imidazole, 20 mM taurine, 50 mM 2-(*N*-morpholino) ethanesulfonic acid hydrate (MES), 0.5 mM dithiothreitol, 6.56 mM MgCl_2_·6H_2_O, 5.77 mM ATP, and 15 mM phosphocreatine (pH 7.1).

A blinded assessor, who did not know the origin of the muscle specimens or the participants’ diagnoses, evaluated the muscle specimens. After careful dissection of each muscle sample using forceps, fibers were chemically permeabilized via incubation in 2 ml of BIOPS containing saponin (50 μg/ ml) for 30 min [37]. Muscle fibers were subsequently incubated for 10 min at 4 °C in ice-cold mitochondrial respiration medium (MiR06; 0.5 mM EGTA, 3 mM MgCl_2_, 60 mM K-lactobionate, 20 mM taurine, 10 mM KH_2_PO_4_, 20 mM HEPES, 110 mM sucrose, and 1 g/l bovine serum albumin essentially fatty acid free, adjusted to pH 7.1, 2800 units/mg solid catalase lypophilized powder). The fibers’ wet weight was measured on a microbalance (Mettler Toledo, Greifensee, Switzerland).

Each biopsy specimen was evaluated for visual quality (based on a scale of 1–5, with 1 = poor and 5 = excellent) and for quantity (based on wet weight in mg).

### High-resolution respirometry

A blinded assessor performed high-resolution respirometry on the muscle specimens and the related data collection and analysis. Measurements of oxygen consumption were carried out at 37 °C using the 2-chamber titration-injection respirometer Oxygraph-2 k (Oroboros Instruments, Innsbruck, Austria). All experiments were carried out in a hyperoxygenated chamber to prevent any potential oxygen diffusion limitation [37]. Oxygen concentration (μM = nmol/ml) and oxygen flux (pmol.s^− 1^.mg^− 1^; negative time derivative of oxygen concentration, divided by muscle wet weight) were recorded using DatLab software (Oroboros Instruments). For the substrate-uncoupler-inhibitor titration protocol, the following substrates were added (as final concentrations):

- Malate (2 mM) and glutamate (10 mM) to support leak respiration without adenylates (LEAK, L_N_).
- Active respiration was stimulated by addition of adenosine diphosphate (2.5 mM) and pyruvate (5 mM) yielding complex I (CI)-supported oxidative phosphorylation (OXPHOS) capacity (CI_P_).
- After titration of carbonyl cyanide p-(trifluoromethoxy) phenylhydrazone (FCCP; a total of 1.5 μM in steps of 0.5 μM) electron transfer capacity (ETC) of CI (CI_E_) was recorded.
- Subsequently, succinate (10 mM) was added to stimulate maximal ETC of CI and CII (CI + II_E_).
- Finally, rotenone (0.5 μM) was added to inhibit CI, yielding ETC of CII (CII_E_) and antimycin A (2.5 μM) and malonic acid (5 mM) to yield residual oxygen consumption (ROX).

### Statistical analysis

Data were extracted from the DatLab-program and compiled into a spreadsheet. SPSS for Windows (SPSS, 2009, Chicago, IL) was used for subsequent statistical analysis. Data were checked for normal distribution by Kolmogorov-Smirnov test, depending on the distribution. Baseline and endpoint data were analyzed using descriptive statistics. The difference between the mean values of the different muscle groups was assessed by a one-way analysis of variance. The significance level was set at *p* ≤ 0.01; *p* ≤ 0.05 and of *p* ≤ 0.1 were considered as trends. Data are presented as mean ± standard deviation (SD). Because this is an explorative study, no correction for multiple testing was applied. There were no previous data available from the literature to perform a sample size calculation for this pilot study. Analysis was performed on a per-protocol basis.

## Results

The baseline demographic, anthropometric and clinical characteristics of the participants were similar for both groups and are summarized in Table 1.

**Table 1:** Comparison of baseline characteristics of gluteus medius and descending trapezius myofascial trigger point (MTrP) groups

Parameter	Gluteus Medius MTrP (*n* = 10)	Descending Trapezius MTrP (*n* = 10)
Age (y)	38.7 ± 5.1 (21–42)	37.6 ± 6.2 (31–45)
Pain intensity NRS	6.8 ± 1.2	6.2 ± 1.5
Weight (kg)	81.2 ± 17.5	86.2 ± 12.8
Height (m)	1.8 ± 0.08	1.8 ± 0.06
Smoker, n (%)	7 (70%)	6 (60%)
Body Mass Index (kg/m^2^)	26.1 ± 3.6	24.9 ± 4.2
Physical activities/sports (minutes/week)	216 ± 154.8	185 ± 90.6
Laboratory tests		
C-reactive protein (mg.dl^−1^)	0.11 ± 0.12	0.15 ± 0.25
Creatine kinase (U.l^− 1^)	222.5 ± 161.3	204.6 ± 128.5
Lactate dehydrogenase (U.l^− 1^)	182.1 ± 26.9	186.3 ± 38.0
Prothrombin time (%)	101.9% ± 11.7	107.1 ± 11.9

Values are mean ± SD; *NRS* Numeric Rating Scale

**Fig. 1:**
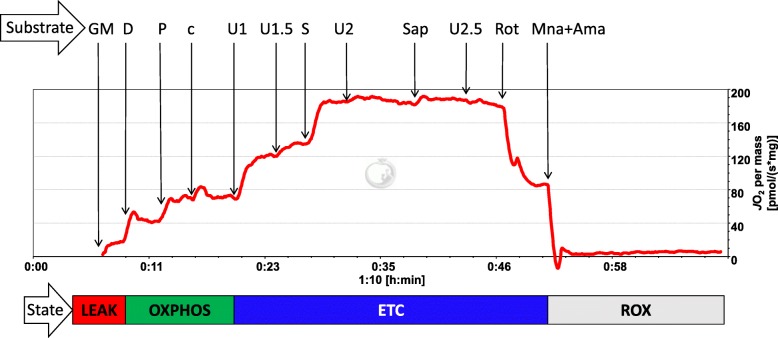
High-resolution respirometry with permeabilized fibers from a muscle biopsy sample. Oxygen flux (*J*O_2_) is displayed as pmol O_2_.s^− 1^.mg^− 1^ wet weight and changes in response to application of the following substrate-uncoupler-inhibitor titration protocol: mitochondrial leak state without adenylates (L_N_) after addition of glutamate (G) and malate (M), complex I-supported oxidative phosphorylation capacity (OXPHOS) after addition of ADP (D), pyruvate (P) and cytochrome c (c), complex I-supported electron transfer capacity (ETC) after addition of an uncoupler (U), and succinate-supported ETC after addition of succinate (S), followed by titration of rotenone (Rot); at the end of the protocol, malonic acid (Mna) and antimycin A were added. Abbreviations: CI_P_ = complex I-supported oxidative phosphorylation capacity; CI_E_ = complex I-supported ETC; CII_E_ = ETC of CII; CI + II_E_ = maximal ETC of CI and CII; ETC = electron transfer capacity; OXPHOS = oxidative phosphorylation; L_N_ = leak state without adenylates

A representative mitochondrial trace of one participant for evaluating mitochondrial function is shown in Fig. 1.

### Quantitative differences in mitochondrial function

**Fig. 2:**
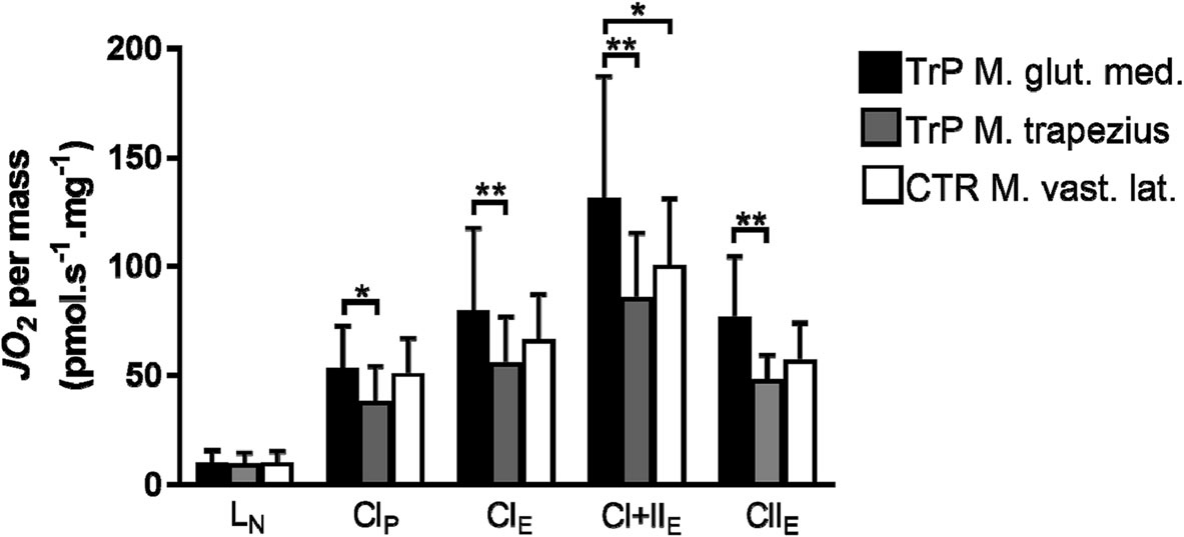
Differences in mass-specific mitochondrial respiration among the different muscle groups. Mass-specific mitochondrial respiration among different muscle groups affected by a myofascial trigger point (m. gluteus medius and m. trapezius) and the unaffected control muscle (m. vastus lateralis) after initiating mitochondrial leak state without adenylates (L_N_), complex I-supported oxidative phosphorylation capacity (CI_P_), complex I-supported electron transfer capacity (ETC) of CI (CI_E_), maximal ETC of CI and CII (CI + II_E_) and ETC of CII (CII_E_). Abbreviations: TrP M. glut. Med. = musculus gluteus medius trigger point; TrP M. trapezius = musculus trapezius trigger point; CTR M. vast. Lat. = musculus vastus lateralis control muscle; see Fig. 1 for additional abbreviations

With the exception of L_N_, mass-specific CI_P_ (53.5 ± 19.3 vs 37.9 ± 6.3 pmol.s^− 1^.mg^− 1^), CI_E_ (79.8 ± 37.6 vs 56.0 ± 20.7 pmol.s^− 1^.mg^− 1^), CI + II_E_ (131.5 ± 55.5 vs 85.9 ± 29.2 pmol.s^− 1^.mg^− 1^) and CII_E_ (76.9 ± 27.6 vs 47.9 ± 11.4 pmol.s^− 1^.mg^− 1^) were all lower (all *p* < 0.05) in the TTP than in the GTP (Fig. 2). CI + II_E_ of the TTP was lower compared to the control m. vastus lateralis (131.5 ± 55.5 vs 100.5 ± 30.8 pmol.s^− 1^.mg^− 1^, p < 0.05). No differences were observed in any respiratory state between the GTP and the control m. vastus lateralis.

### Qualitative differences in mitochondrial function

**Fig. 3:**
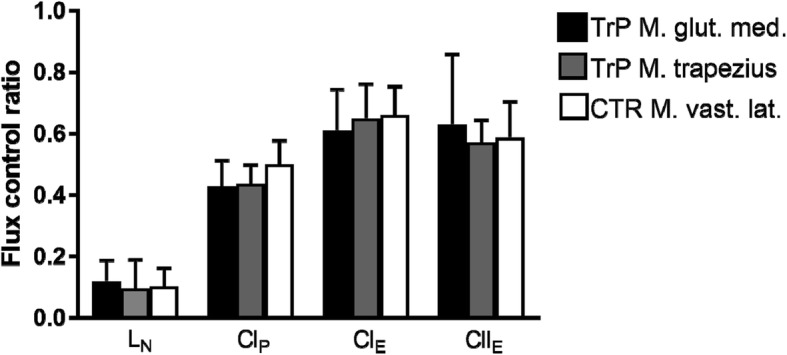
Respiratory states normalized for the internal reference state of electron transfer capacity (ETC). Normalizing respiration for ETC of CI and CII (CI + II_E_) results in flux control ratios, which reflect important mitochondrial qualitative alterations in mitochondrial function. The leak state without adenylates (L_N_), complex I-supported oxidative phosphorylation capacity (CI_P_), complex I-supported ETC (CI_E_), and ETC of CII (CII_E_) are displayed, and all states are normalized to maximal ETC of CI and CII (CI + II_E_). Abbreviations: TrP M. glut. Med. = musculus gluteus medius trigger point; TrP M. trapezius = musculus trapezius trigger point; CTR M. vast. Lat. = musculus vastus lateralis control muscle; see Fig. 1 for additional abbreviations

When normalizing respiratory states for the internal reference state of maximal ETC of CI + II, the resulting FCRs reflect important qualitative alterations in mitochondrial function (Fig. 3). Surprisingly, there were no differences across all groups for FCR, indicating no qualitative differences with regard to mitochondrial function between GTP, TTP, and m. vastus lateralis.

Biopsy assessment, safety and acceptance of the biopsy procedure for all 3 muscle groups are summarized in Table 2. Muscle samples of very good quality and similar yield were obtained from all 3 muscle groups. None of the groups had hematomas or surgical complications. The mean pain reported for the biopsy procedure was higher for the TTP group (1.1 ± 2.3) than for the GTP (0.25 ± 0.35) and control (0.2 ± 0.4), although pain was generally minimal for all 3 groups. For all 3 muscle groups, participants reported a low procedural burden.

**Table 2:** Biopsy yield, quality, safety, and acceptance of biopsy procedure for gluteus medius myofascial trigger point (GTP) and descending trapezius myofascial trigger point (TTP) samples and control (vastus lateralis) samples

	GTP (*n* = 10)	TTP (*n* = 10)	Vastus Lateralis (*n* = 20)
Mean biopsy yield (mg)	3.7 ± 1.6	3.4 ± 2.2	3.8 ± 2.1
Mean microscopic biopsy quality (1–5)^a^	4.9 ± 0.32	4.7 ± 0.48	4.7 ± 0.47
Clinical wound assessment (number of patients without signs for local infection and inflammation), n	10	10	20
Mean ultrasonic volume of hematoma (cm^3^)	0	0	0
Surgical complications, n	0	0	0
Mean pain (NRS) at biopsy location after 7 days	0.25 ± 0.35	1.1 ± 2.3	0.2 ± 0.4
Procedure burden at biopsy location (1–4)^b^	3.2 ± 0.8	3.1 ± 0.99	3.1 ± 0.91

Values are mean ± SD

^a^Microscopic biopsy quality scale of 1–5: 1 = poor, 2 = fair, 3 = good, 4 = very good, and 5 = excellent

^b^Procedure burden scale of 1–4: 1 = very high, 2 = high, 3 = low, and 4 = none

*NRS* Numeric Rating Scale

## Discussion

This pilot study demonstrates the feasibility of a minimally invasive biopsy technique to obtain muscle tissue from an MTrP in sufficient amount and quality for high-resolution respirometry analysis of mitochondrial function. The use of fresh muscle biopsy samples for high-resolution respirometry allows for the direct measurement of oxygen consumption and provides detailed information about mitochondrial functional integrity and energetic capacity (Figs. 2 and 3). Previous histological examination of MTrP biopsies revealed mitochondrial swelling, resulting in reduced ATP concentrations and blood flow and increased metabolic stress that contributed to persistent MTrPs [2]. In the current study, high-resolution respirometry provides evidence that the presence of an MTrP for up to 12 months does not influence mitochondrial function in the corresponding muscle. There were no qualitative differences in mitochondrial function among the MTrP samples and the control samples. Our results suggest that mitochondria do not have role in the development of MTrPs.

The presence of quantitative differences in respiratory capacity, enzymatic equipment, and fiber type distribution between different muscles of the human body is well established [38–40]. It has been shown that mitochondrial density in the arm is half of that in the leg in a cohort of healthy males [40]. It is therefore not surprising that, in the current study, quantitative differences exist with regard to mitochondrial function among the m. gluteus medius, the m. vastus lateralis, and the m. trapezius. In humans, the 2 former muscles are energetically challenged and extensively involved in locomotion, while the trapezius muscle has mainly postural functions with low level sustained muscle activity above resting level. [41]. Mass-specific mitochondrial respiration (expressed per mg of muscle tissue) was highest in m. gluteus medius, followed by m. trapezius and m. vastus lateralis (Fig. 2), whereas mitochondrial respiration normalized to maximal ETC of CI + CII was not different between the different muscles (Fig. 3). Normalization for maximal respiration yields lower and upper limits of 0.0 and 1.0 (0% and 100%). Internal normalization has the advantage of expressing respiratory control independent of mitochondrial content and will hence indicate any qualitative changes within the respiratory system. Our results suggest that changes in mass-specific mitochondrial respiration are mainly the result of changes in mitochondrial content as naturally present between different muscles of the human body.

Until now, it was not known if mitochondria also play a role in the development and manifestation of MTrPs. Our results indicate that qualitative skeletal muscle bioenergetics are not impaired in muscles affected by a trigger point. As our study only involved in vitro analysis, we cannot exclude, however, possible in vivo impairments of mitochondrial function. Based on our results, we assume that alterations in mitochondrial function do not play a major role in the development of trigger points, at least up to 12 months after diagnosis.

It will be challenging to identify the point at which mitochondrial function is possibly impaired in the affected muscle. However, this is clinically important, as interventions at the point where impaired mitochondrial function is still reversible will prevent disease progression to a level where mitochondrial function is irreversibly damaged. It therefore remains highly relevant to study mitochondrial function and its relation to trigger point development and progression.

Although not intended as therapeutic intervention, the diagnostic biopsy procedure resolved the reported pain intensity in almost all patients. This response is similar to dry needling interventions for myofascial trigger points [6]. There is a significant bias in the assessment of pain levels at baseline and 1 week after the biopsy procedure in our study. Pain intensity was assessed pre biopsy by palpation and pressure applied to the trigger point. One week post biopsy, only spontaneous reported pain intensity was documented. The authors wanted to reduce patient discomfort and possible surgical wound-related complications. By choosing a later time point in future studies, this bias can be eliminated. In the current study, pain reduction was not an intended outcome measure, therefor pain assessment was not identical at both time points. This pilot study, being exploratory in nature, was limited by its sample size comprising a homogeneous, younger, male population. MTrPs are more prevalent in women and elderly individuals [2, 12], and impaired mitochondrial function is also more prevalent in older populations [42–45]. A large-scale clinical trial including women and older adults is necessary to confirm our findings.

A further limitation of our study is the lack of a clear presentation of clinical data. One inclusion criteria was the documentation of duration of trigger point-related pain complaint. Patients were included into the study if pain existed more than one and less than twelve months, without documenting the exact duration.

This study did assess pain related to the biopsy procedure, but these data were not collected during/immediately after the procedure. Therefore, the findings related to the acceptability of the procedure are limited in terms of pain.

## Conclusions

This pilot study used a minimally invasive and safe technique for obtaining biopsies from MTrPs suitable for high-resolution respirometry analysis of mitochondrial function in MTrPs. The results suggest that there are no qualitative differences with regard to mitochondrial function in biopsies of MTrPs of the m. trapezius and m. gluteus medius muscles compared to control biopsies of the vastus lateralis muscle, therefore implying that alterations of mitochondrial function do not appear to have a role in the development of MTrPs, at least up to 12 months after diagnosis.

## Abbreviations

31P-MRS: Phosphorus-31 magnetic resonance spectroscopy
ATP: adenosine triphosphate
BIOPS: biopsy preservation solution
BMI: Body-Mass Index
CI + II_E_: maximal ETC of CI and CII
CI: Complex I
CII: Complex II
CII_E_: ETC of CII
CI_P_: Complex I-supported oxidative phosphorylation capacity
ETC: electron transfer capacity
FCR: flux control ratio
GTP: gluteus medius myofascial trigger point
IL: interleukin
L_N_: leak state without adenylates
m.: musculus
mtDNA: mitochondrial DNA, the mitochondrial genome
MTrP: myofascial trigger point
NRS: Numeric Rating Scale
OXPHOS: oxidative phosphorylation
PCr: phosphocreatine
PI: principal investigator
ROS: reactive oxygen species
TTP: descending trapezius myofascial trigger point
